# Major Transcriptome Reprogramming Underlies Floral Mimicry Induced by the Rust Fungus *Puccinia monoica* in *Boechera* *stricta*

**DOI:** 10.1371/journal.pone.0075293

**Published:** 2013-09-17

**Authors:** Liliana M. Cano, Sylvain Raffaele, Riston H. Haugen, Diane G. O. Saunders, Lauriebeth Leonelli, Dan MacLean, Saskia A. Hogenhout, Sophien Kamoun

**Affiliations:** 1 The Sainsbury Laboratory, Norwich, United Kingdom; 2 Laboratoire des Interactions Plantes Micro-organismes, UMR441 INRA - UMR2594 CNRS, Castanet Tolosan, France; 3 Black Hills State University, Integrative Genomics Program, Spearfish, South Dakota, United States of America; 4 The Department of Plant and Microbial Biology, University of California, Berkeley, California, United States of America; 5 Cell and Developmental Biology, The John Innes Centre, Norwich Research Park, Norwich, United Kingdom; University of Wisconsin - Madison, United States of America

## Abstract

*Pucciniamonoica* is a spectacular plant parasitic rust fungus that triggers the formation of flower-like structures (pseudoflowers) in its Brassicaceae host plant *Boechera* *stricta* . Pseudoflowers mimic in shape, color, nectar and scent co-occurring and unrelated flowers such as buttercups. They act to attract insects thereby aiding spore dispersal and sexual reproduction of the rust fungus. Although much ecological research has been performed on *P* *. monoica* -induced pseudoflowers, this system has yet to be investigated at the molecular or genomic level. To date, the molecular alterations underlying the development of pseudoflowers and the genes involved have not been described. To address this, we performed gene expression profiling to reveal 256 plant biological processes that are significantly altered in pseudoflowers. Among these biological processes, plant genes involved in cell fate specification, regulation of transcription, reproduction, floral organ development, anthocyanin (major floral pigments) and terpenoid biosynthesis (major floral volatile compounds) were down-regulated in pseudoflowers. In contrast, plant genes involved in shoot, cotyledon and leaf development, carbohydrate transport, wax biosynthesis, cutin transport and L-phenylalanine metabolism (pathway that results in phenylethanol and phenylacetaldehyde volatile production) were up-regulated. These findings point to an extensive reprogramming of host genes by the rust pathogen to induce floral mimicry. We also highlight 31 differentially regulated plant genes that are enriched in the biological processes mentioned above, and are potentially involved in the formation of pseudoflowers. This work illustrates the complex perturbations induced by rust pathogens in their host plants, and provides a starting point for understanding the molecular mechanisms of pathogen-induced floral mimicry.

## Introduction

Many phytopathogens have evolved the ability to manipulate host plants to acquire nutrients and evade host defenses. These include microbes that possess the ability to cause dramatic morphological and physiological changes in their hosts. These changes can even lead to behavioral manipulation of a third organism, which is often an insect vector. Organisms with this "long-reach" phenotype include several species of obligate plant pathogenic bacteria and fungi. One example is the Aster Yellows phytoplasma strain Witches’ Broom (AY-WB), which infects a broad range of plant hosts [1,2] and induces a variety of morphological changes. These include the conversion of floral organs into leaves (phyllody), clustering of stems and branches (witches’ broom), green pigmentation of non-green flower tissues (virescence) and growth of elongated stalks (bolting) [1]. These morphological changes are thought to entice egg-laying insects to visit infected plants where they feed on contaminated tissue and transmit bacteria to new host plants [3,4]. Another example is the rust fungus *Pucciniamonoica* that manipulates its plant host *Boechera* *stricta* (syn. *Arabis* *drummondii* ) to create elaborate pseudoflowers. These structures are completely novel to the plant’s native architecture [5] and act to lure pollinators from co-blooming plant species by offering olfactory incentives and a sugary reward [5]. Pollinator visits are essential for the completion of the sexual reproductive cycle of the fungus as they transfer spores of opposite mating types between pseudoflowers [5].

Despite recent advances in our understanding of the molecular mechanisms underlying pathogen-derived host manipulation, little is known regarding the transcriptional changes that occur in plants upon infection by pathogens that cause developmental reprogramming. To address this, we examined the effect of *P* *. monoica* infection on its host plant *B* *. stricta* , a close relative of *Arabidopsis* . *B* *. stricta* belongs to the Brassicaceae and grows mainly in the alpine regions of western North America [6]. In late summer, wind-borne basidiospores of *P* *. monoica* , produced on an unknown primary host grass, systematically infect the apical meristem of *B* *. stricta* , its secondary host [5,7]. *P* *. monoica* infection inhibits flowering and radically transforms *B* *. stricta* morphology, manipulating it to produce yellow flower-like structures that mimic true flowers of the unrelated co-blooming buttercups, *Ranunculus* *inamoenus* [8,9]. Although these pseudoflowers are visually similar in size, shape, color, and nectar production to true buttercup flowers, they produce a distinct sweet fragrance that attracts insect visitors [8,10,11]. Due to the heterothallic nature of *P* *. monoica* , these spore-laden insect visitors are critical to completion of the pathogens life cycle [5,12].

*B* *. stricta* is a close relative of the model plant *Arabidopsis thaliana*, therefore we can utilize the extensive genomic resources available for *A. thaliana* to study *P* *. monoica* - *B* *. stricta* interactions [13,14]. To this aim, we employed *A. thaliana* whole-genome microarrays to analyze transcriptional changes in *B* *. stricta* gene expression upon *P* *. monoica* infection. We used a NimbleGen microarray to determine the expression levels of transcripts isolated from *P* *. monoica* -induced pseudoflowers (‘Pf’), uninfected *B* *. stricta* flowers (‘F’), and uninfected *B* *. stricta* stems and leaves (‘SL’). To compare relative gene expression levels we used Rank Products (RP) protocols [15]. This analysis identified 1036 and 910 genes that showed significant changes in expression in comparisons between ‘Pf’ vs. ‘SL’ and ‘F’ vs. ‘SL’ respectively. Next, we performed an enrichment analysis of Gene Ontology terms describing Biological Processes (GOBP) using BiNGO in Cytoscape on these gene sets [16]. We found a total of 256 and 199 GOBP terms significantly enriched in ‘Pf’ vs. ‘SL’ and ‘F’ vs. ‘SL’ comparisons, respectively. Among 256 key biological processes, we identified 31 gene candidates (20 up-regulated, and 11 down-regulated) showing significant alterations in expression between pseudoflowers ‘Pf’ and uninfected *B* *. stricta* stems and leaves ‘SL’. These included genes involved in (i) leaf, stem and flower development, (ii) organ symmetry, (iii) metabolism of sugars, (iv) transport of sugars and lipids, and (v) wax and volatiles synthesis.

Our findings point to major reprogramming of the *B* *. stricta* transcriptome during infection, with several key biological processes acting as targets that could account for *P* *. monoica* -induced pseudoflower formation. This study is a crucial step towards understanding how this rust fungus manipulates its host plant at the molecular level and how such “long-reach” pathogens act to indirectly manipulate insect vectors to achieve sexual reproduction.

## Results and Discussion

### Gene expression profiling of pseudoflowers

**Figure 1:**
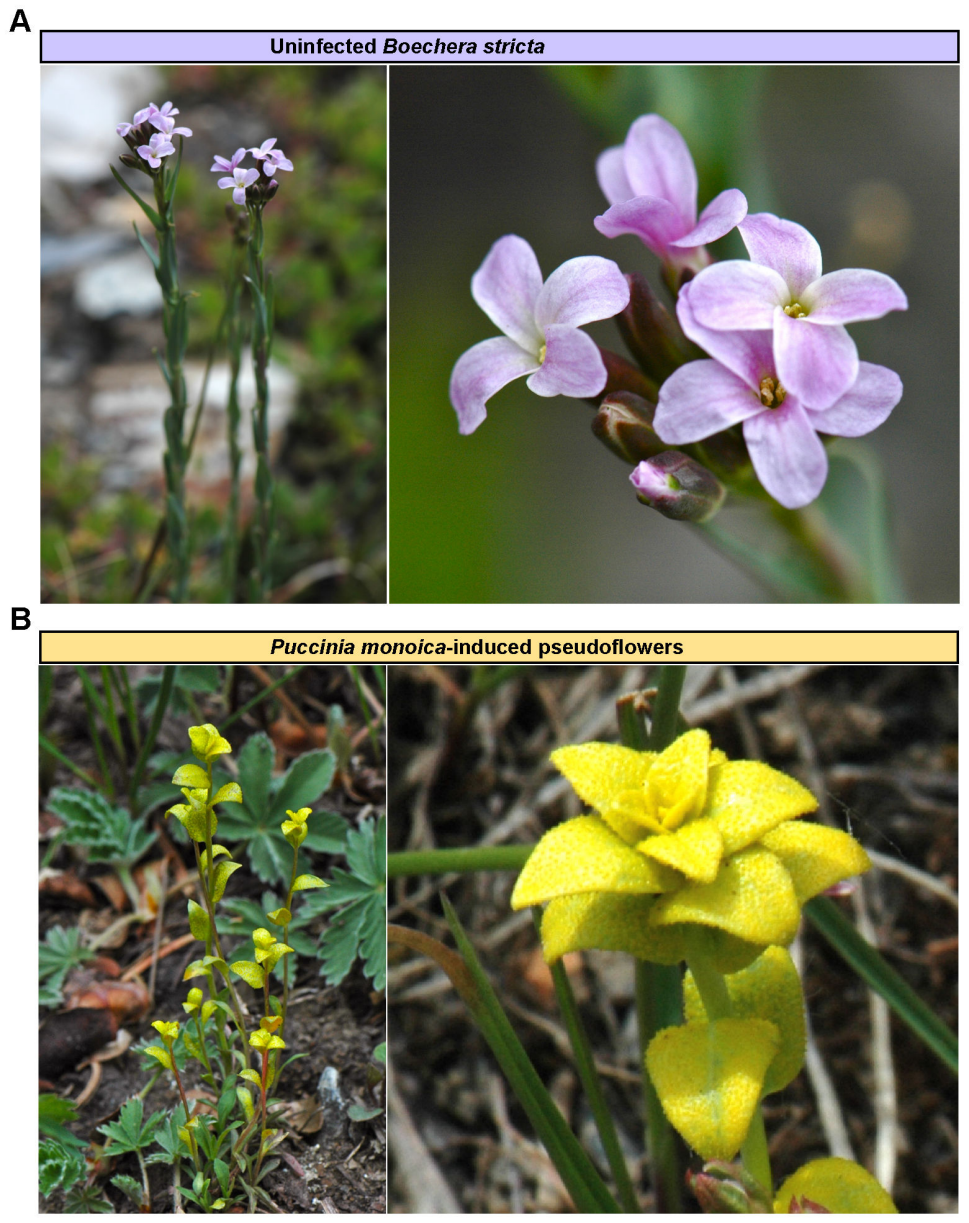
Illustration of floral mimicry produced by the pseudoflower-forming rust fungus *Pucciniamonoica* . (**A**) Picture of uninfected flowering *Boechera* *stricta* plant (left) and a close up picture of its light pink flowers (right). (**B**) Pictures of vegetative tissues of *B* *. stricta* plants that produce pseudoflowers upon infection with *Pucciniamonoica* (left) and a close up of a yellow *P* *. monoica* pseudoflower (right). Samples from *B* *. stricta* (**A**) and pseudoflowers (**B**) were collected near Gunnison, Colorado, United States of America.

**Figure 2:**
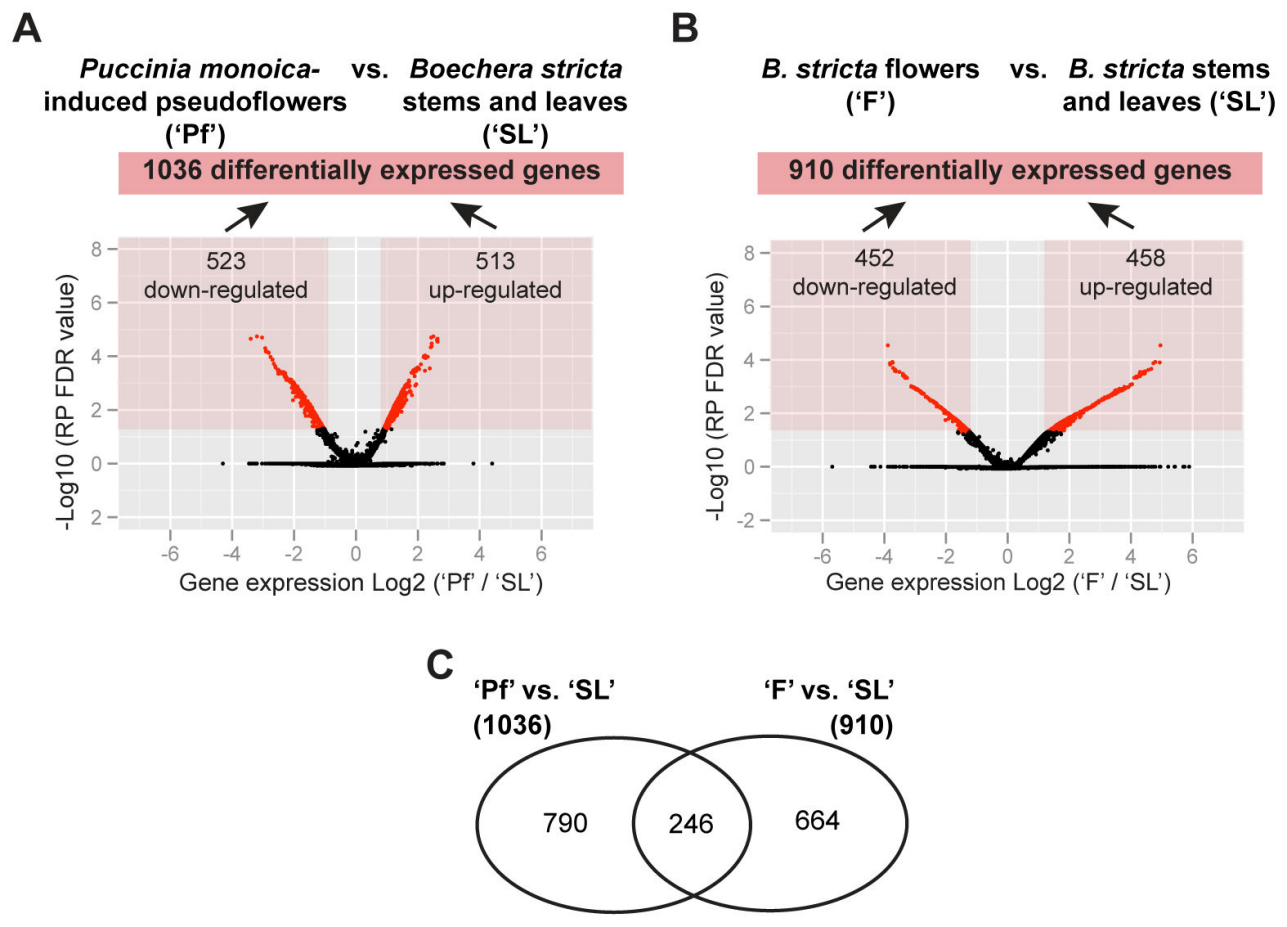
Differentially expressed genes in pseudoflowers and uninfected *Boechera* *stricta* flowers using rank products (RP) analysis. (**A**) Volcano plots showing changes in gene expression in *Pucciniamonoica* -induced pseudoflowers (‘Pf’) vs. uninfected *Boechera* *stricta* plant stems and leaves (‘SL’). (**B**) Volcano plots showing changes in gene expression in uninfected *B* *. stricta* flowers (‘F’) vs. uninfected *B* *. stricta* stems and leaves (‘SL’). Each point in the volcano plot represents changes in gene expression from a single *Arabidopsis thaliana* gene. Red points indicate genes that are significantly up or down-regulated with a RP FDR value < 0.05. X-axis correspond the log_2_ ratio (‘Pf’/’SL’ or ‘F’/’SL’ comparison) and the y-axis correspond to the –log_10_ of RP FDR value. (**C**) Venn diagram showing number of genes that are differentially regulated specifically in ‘Pf’ vs. ‘SL’ and ‘F’ vs. ‘SL’ comparisons.

To identify changes in *Boechera* *stricta* gene expression in *Pucciniamonoica* -induced pseudoflowers we hybridized cDNA to a customized NimbleGen expression array covering all predicted coding genes in the *Arabidopsis thaliana* genome, a close relative of *B* *. stricta* . Samples were collected as follows: (i) uninfected plant stems and leaves (‘SL’), (ii) uninfected plant flowers (‘F’) (Figure 1A) and (iii) pseudoflowers from *P* *. monoica* -infected plants (‘Pf’) (Figure 1B). Using a Rank Products (RP) analysis, we identified 1036 genes (Table S1) and 910 genes (Table S2) showing significant differential expression in the ‘Pf’ vs. ‘SL’ and ‘F’ vs. ‘SL’ comparisons, respectively (Figure 2A-B). Among these, a total of 790 genes showed differential expression in the ‘Pf’ vs. ‘SL’ comparison alone, and 664 showed differential expression only in the ‘F’ vs. ‘SL’ comparison (Figure 2C).

RP analysis generated two RP FDR values for each gene indicating the probability of being up or down-regulated [15]. Of the 1036 genes present in the ‘Pf’ vs. ‘SL’ comparison, we determined 513 to be up-regulated and 523 to be down-regulated (with RP FDR values < 0.05) (Figure 2A and Table S1). In the ‘F’ vs. ‘SL’ comparison, out of the pool of 910 differentially expressed genes, 458 genes were up-regulated and 452 down-regulated (Figure 2B and Table S2).

### Validation of a subset of genes differentially regulated in pseudoflowers by qRT-PCR

**Figure 3:**
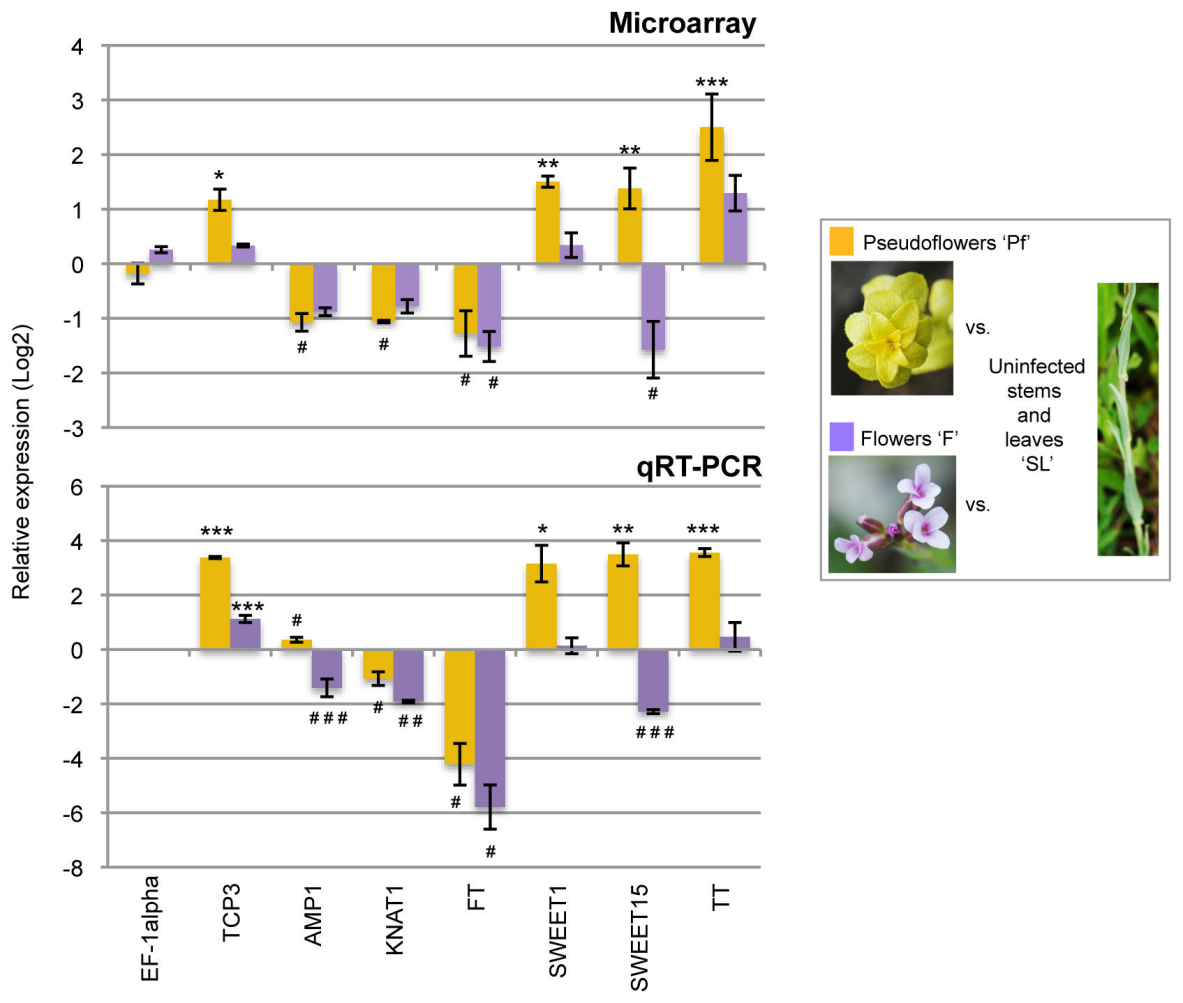
qRT-PCR validation of differentially expressed genes in pseudoflowers. Quantitative Real Time PCR (qRT-PCR) on a panel of seven genes was used to verify the transcriptional changes observed by microarray analysis. Consistent with the microarray results, expression of *TEOSINTE* *BRANCHED1, CYCLOIDEA, and* *PCF* *TRANSCRIPTION* *FACTOR3* (*TCP3*, At1g53230), *SUGAR* *TRANSPORTER1* (*SWEET1*, At1g21460), *SUGAR* *TRANSPORTER15* (*SWEET15*, At5g13170) and *TYROSINE* *TRANSAMINASE* enzyme encoding gene (*TT*, At4g23590) genes was up-regulated in *Pucciniamonoica* -induced pseudoflowers (‘Pf’) compared to *B* *. stricta* stems and leaves (‘SL’), while *ALTERED* *MERISTEM* *PROGRAMMING1* (*AMP1*, At3g54720), *KNOTTED-LIKE1* (*KNAT1*, At4g08150) and *FLOWERING* *LOCUS* *T* (*FT*, At1g65480) genes was down-regulated (see Table 1). In addition, *SWEET15* and *FT* genes were confirmed to be down-regulated in uninfected *B* *. stricta* flowers (‘F’) compared to ‘SL’ as shown by microarray analysis (see Table S2). To indicate the mode of regulation we used two symbols: ‘*’ for significant up-regulation and ‘^#^’ for significant down-regulation. The number of symbols indicates level of significance: one for *P* < 0.05, two for *P* < 0.01 and three for *P* < 0.001. The error bars represents standard error of the mean.

To test for the robustness of the gene regulation patterns we observed by microarrays analysis, we validated by qRT-PCR the expression of a subset of seven genes that are differentially regulated in *Pucciniamonoica* -induced pseudoflowers (‘Pf’) compared to uninfected *Boechera* *stricta* stems and leaves (‘SL’) (Figure 3). We selected *TEOSINTE BRANCHED1, CYCLOIDEA, and PCF TRANSCRIPTION FACTOR3 TCP3* (At1g53230), *ALTERED MERISTEM PROGRAMMING1 AMP1* (At3g54720), *KNOTTED-LIKE1 KNAT1* (At4g08150) and *FLOWERING LOCUS T FT* (At1g65480) genes as representatives of host cell developmental processes altered in pseudoflowers (‘Pf’) such leaf morphogenesis, pattern and cell specification, respectively (Table 1, see discussion below). Also, we selected *SUGAR TRANSPORTER1* (*SWEET1*, At1g21460) and *SUGAR TRANSPORTER15* (*SWEET15*, At5g13170) genes involved in carbohydrate transport and *TYROSINE TRANSAMINASE* enzyme-encoding gene (*TT*, At4g23590) that participates in organic volatile compounds synthetic pathway (Table 1). These seven genes were also selected as examples of the different modes of regulation in gene expression observed in ‘Pf’. *TCP3*, *SWEET1*, *SWEET15* and *TT* genes are up-regulated in pseudoflowers whereas *AMP1*, *KNAT1* and *FT* are down-regulated and these observations support the expression patterns found by microarray analysis (Figure 3). Even though small differences were found in the strength of the changes of gene expression in pseudoflowers compared to uninfected *B* *. stricta* stems and leaves, the overall expression patterns from qRT-PCR were very similar and matches the data obtained from the microarray analysis (Figure 3).

**Table 1:** *Arabidopsis thaliana* homologs of *Boechera* *stricta* genes with altered expression in pseudoflowers.

**Gene ID**	**Gene name**	**Common name**	**Classification used in this study**	**Expression in pseudoflowers^a^**	**Log_2_**	**RP FDR value^b^**	**GOBP^c^**	**GOBP description^c^**
At4g18390	*TEOSINTE BRANCHED1, CYCLOIDEA, and PCF TRANSCRIPTION FACTOR2*	*TCP2*	De-differentiation of infected mesophyll cells	Up-regulated	1.25	9.44E-03	9965	Leaf morphogenesis
At1g53230	*TEOSINTE BRANCHED1, CYCLOIDEA, and PCF TRANSCRIPTION FACTOR3*	*TCP3*	De-differentiation of infected mesophyll cells	Up-regulated	1.17	1.38E-02	9965	Leaf morphogenesis
At3g54720	*ALTERED MERISTEM PROGRAMMING1*	*AMP1*	De-differentiation of infected mesophyll cells	Down-regulated	-1.07	4.21E-02	7389	Pattern specification process
At2g29125	*ROTUNDIFOLIA-LIKE2*	*RTFL2*	Alteration of the rate of cell proliferation	Up-regulated	1.44	3.39E-03	48367	Shoot development
At1g13710	*CYTOCHROME P450 MONOOXYGENASE*	*CYP78A5*	Alteration of coordinated organ growth and symmetry	Up-regulated	1.02	3.86E-02	48366	Leaf development
At2g45190	*FILAMENTOUS FLOWER*	*FIL*	Alteration of vascular patterning and phyllotaxy	Up-regulated	2.23	9.33E-05	10158	Abaxial cell fate specification
At1g01030	*NGATHA3*	*NGA3*	Alteration of vascular patterning and phyllotaxy	Up-regulated	1.17	1.11E-02	48367	Shoot development
At1g30490	*PHAVOLUTA*	*PHV*	Alteration of vascular patterning and phyllotaxy	Down-regulated	-1.07	4.22E-02	10051	Xylem and phloem pattern formation
At1g52150	*INCURVATA4*	*ICU4*	Alteration of vascular patterning and phyllotaxy	Down-regulated	-1.12	3.27E-02	10051	Xylem and phloem pattern formation
At3g07970	*QUARTER2*	*QRT2*	Inhibition of flower differentiation and maturation	Up-regulated	0.93	4.14E-02	48869	Cellular developmental process
At4g08150	*KNOTTED-LIKE1*	*KNAT1*	Inhibition of flower differentiation and maturation	Down-regulated	-1.06	4.54E-02	1708	Cell fate specification
At2g27990	*POUND-FOOLISH*	*PNF*	Inhibition of flower differentiation and maturation	Down-regulated	-1.18	2.93E-02	10076	Maintenance of floral meristem identity
At1g65480	*FLOWERING LOCUS T*	*FT*	Inhibition of flower differentiation and maturation	Down-regulated	-1.28	2.65E-02	3	Reproduction
At2g03710	*SEPATALLA4*	*SEP4*	Inhibition of flower differentiation and maturation	Down-regulated	-1.40	9.58E-03	48437	Floral organ development
At4g37390	*INDOLE-3-ACETIC ACID-AMIDO SYNTHASE2*	*GH3.2*	Alteration of auxin homeostasis	Up-regulated	4.40	0.00E+00	9725	Response to hormone stimulus
At1g59500	*INDOLE-3-ACETIC ACID-AMIDO SYNTHASE4*	*GH3.4*	Alteration of auxin homeostasis	Up-regulated	2.64	2.86E-05	9725	Response to hormone stimulus
At1g70560	*TRYPTOPHAN AMINOTRANSFERASE OF ARABIDOPSIS1*	*TAA1*	Alteration of auxin homeostasis	Up-regulated	1.47	4.72E-03	48825	Cotyledon development
At3g14370	*SERINE/THREONINE KINASE*	*WAG2*	Alteration of auxin homeostasis	Up-regulated	1.09	2.12E-02	48825	Cotyledon development
At4g25960	*P-GLYCOPROTEIN2*	*PGP2*	Alteration of auxin homeostasis	Up-regulated	1.04	2.59E-02	55085	Transmembrane transport
At1g51460	*ATP-BINDING-CASSETTE (ABC*)* TRANSPORTER SUPERFAMILY G13*	*ABCG13*	Activation of wax biosynthesis and cutin transport	Up-regulated	2.80	0.00E+00	6869	Lipid transport
At2g15090	*3-KETOACYL-COA SYNTHASE8*	*KCS8*	Activation of wax biosynthesis and cutin transport	Up-regulated	1.31	7.20E-03	6633	Fatty acid biosynthesis
At5g12420	*WAX ESTER SYNTHASE/ACYLCOA: DIACYLGLYCEROL ACETYLTRANSFERASE7*	*WSD7*	Activation of wax biosynthesis and cutin transport	Up-regulated	0.97	4.51E-02	10025	Wax biosynthesis
At5g23940	*CUTICULAR RIDGES*	*DCR*	Activation of wax biosynthesis and cutin transport	Up-regulated	0.94	4.48E-02	6633	Fatty acid biosynthesis
At3g13790	*CELL WALL INVERTASE1*	*cwINV1*	Subversion of sugar metabolism	Up-regulated	2.44	4.29E-05	6950	Response to stress
At1g21460	*SUGAR TRANSPORTER1*	*SWEET1*	Subversion of sugar metabolism	Up-regulated	1.50	1.99E-03	34219	Carbohydrate transmembrane transport
At5g13170	*SUGAR TRANSPORTER15*	*SWEET15*	Subversion of sugar metabolism	Up-regulated	1.38	5.09E-03	34219	Carbohydrate transmembrane transport
At1g68130	*INDETERMINANT DOMAIN14*	*IDD14*	Subversion of sugar metabolism	Down-regulated	-1.18	2.67E-02	45449	Regulation of transcription
At3g43190	*SUCROSE SYNTHASE4*	*SUS4*	Subversion of sugar metabolism	Down-regulated	-2.32	4.39E-04	16051	Carbohydrate biosynthesis
At4g23590	*TYROSINE TRANSAMINASE*	*TT*	Alteration of volatile organic compounds synthesis	Up-regulated	2.50	1.82E-05	6558	L-phenylalanine metabolism
At2g24210	*TERPENE SYNTHASE10*	*TPS10*	Alteration of volatile organic compounds synthesis	Down-regulated	-2.22	7.44E-04	16099	Monoterpenoid biosynthesis
At5g23960	*TERPENE SYNTHASE21*	*TPS21*	Alteration of volatile organic compounds synthesis	Down-regulated	-2.65	1.90E-04	16099	Monoterpenoid biosynthesis

a Expression in *Pucciniamonoica* -induced pseudoflowers (‘Pf’) relative to uninfected *Boechera* *stricta* stems and leaves (‘SL’).

b Rank Product (RP) False Discovery Rate (FDR) values used to estimate differentially expressed genes in *Pucciniamonoica* -induced pseudoflowers (‘Pf’) compared to uninfected *Boechera* *stricta* stems and leaves (‘SL’). Genes with RP FDR value < 0.05 are considered significant.

^c^ Gene ontology terms describing biological processes (GOBP) from *Arabidopsis thaliana* TAIR database version 10 [71].

### Biological processes altered in pseudoflowers

**Figure 4:**
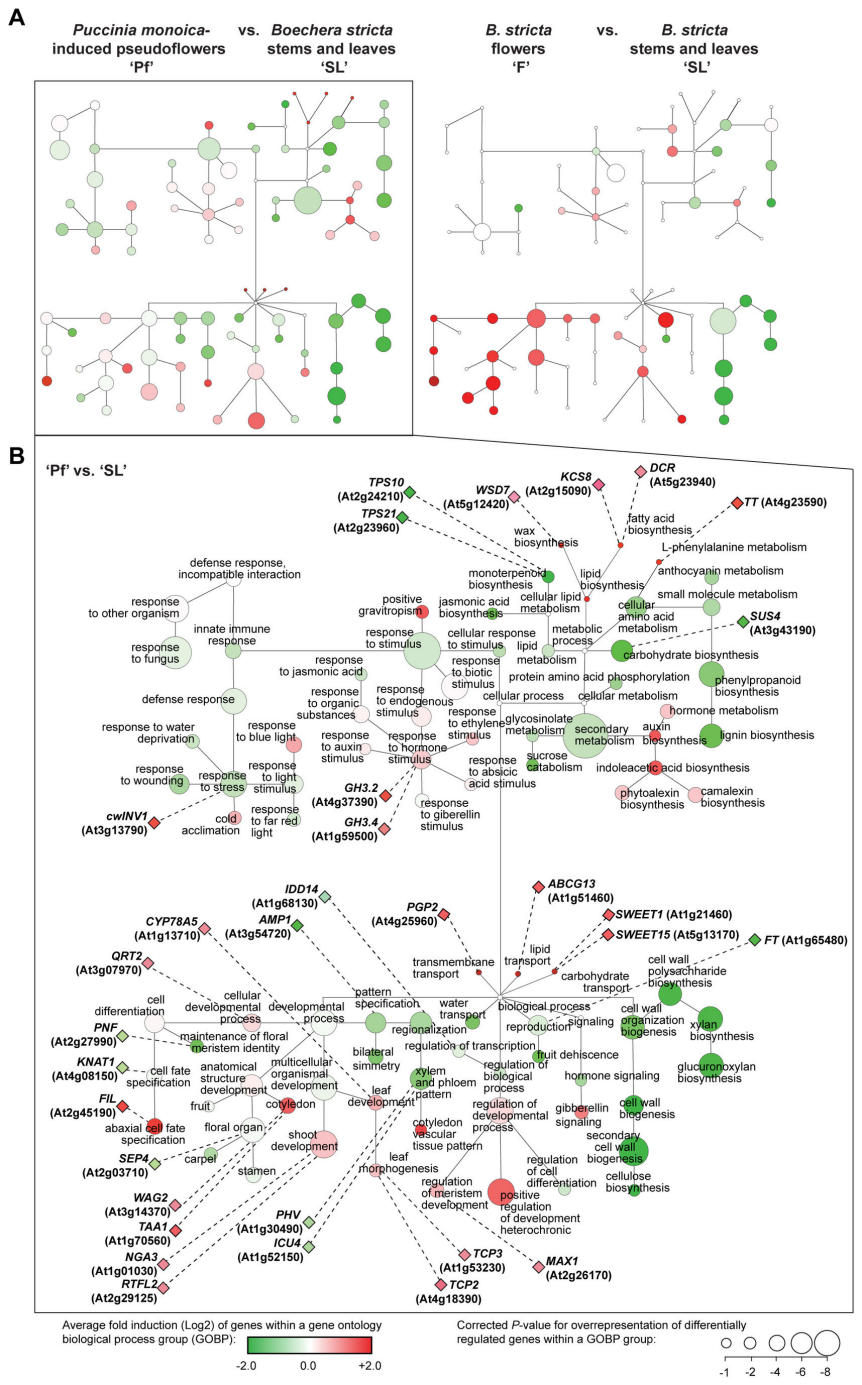
Overview of biological processes altered in pseudoflowers. (**A**) Simplified gene ontology biological processes (GOBP) network showing processes enriched among genes with expression altered in *Pucciniamonoica* -induced pseudoflowers (‘Pf’) compared to uninfected *Boechera* *stricta* stems and leaves (’SL’) and *B* *. stricta* flowers (‘F’) compared to ‘SL’. Node size with average GOBP fold induction (average of log_2_ ratios of all genes within a GOBP in ‘Pf’/’SL’ and ‘F’/’SL’, respectively) from green for average induction folds < 0 that indicates down-regulation to red for average induction folds > 0 that indicates up-regulation (node color). Some nodes and edges have been omitted for clarity. (**B**) Detailed GOBP network showing processes enriched among genes with expression altered in ‘Pf’ vs. ‘SL’. Genes highlighted in the text are indicated with diamonds connected to dashed lines to the processes they are involved in. This network shows same topology as in (**A**).

To identify and annotate biological processes altered during the formation of pseudoflowers, we performed Gene Ontology enrichment analysis for terms describing Biological Processes (GOBP). We identified 256 (Table S3) and 199 (Table S4) GOBP terms significantly enriched in the ‘Pf’ vs. ‘SL’ and ‘F’ vs. ‘SL’ comparisons, respectively. These GOBPs for both comparisons are shown in Figure 4A in a network map as circular nodes that are color-coded according to the average expression (red for up-regulated and green for down-regulated).

*P* *. monoica* -infected plants develop an elongated stem with modified leaves instead of flowers [5]. In accordance, we identified genes involved in maintenance and development of floral organs amongst those down-regulated in *P* *. monoica* -induced pseudoflowers. Functional groups overrepresented among the genes down-regulated specifically in the ‘Pf’ vs. ‘SL’ comparison, but not in the ‘F’ vs. ‘SL’ comparison, included: (1) reproduction (GO:0000003), (2) floral organ development (GO:0048437), (3) carpel development (GO:0048440), (4) stamen development (GO:0048443), (5) cell fate specification (GO:0001708), (6) maintenance of floral meristem identity (GO:0010076), (7) anthocyanin biosynthesis (GO: 00009718), (8) water transport (GO:0006833), (9) pattern specification (GO:0007389), (10) xylem and phloem pattern formation (GO: 0010051), (11) regulation of transcription (GO:0045449), and (12) monoterpenoid biosynthesis (G0: 0016099) (Figure 4B). Among these processes we highlight the down-regulation of monoterpenoid biosynthetic genes (Figure 4B), which is consistent with the observation that *P* *. monoica* induces the synthesis of chemical attractants unrelated to the native floral scent production of the host [5,10]. The distinct fragrance of pseudoflowers contains both phenylacetaldehyde and phenylethanol; compounds that are chemically different to the terpenoids produced in uninfected flowers, but possess the same ability to efficiently attract pollinators [5,10].

Functional groups over-represented and specifically up-regulated in the ‘Pf’ vs. ‘SL’ comparison included (1) shoot development (GO:0048367) (2), cotyledon development (GO:0048825) (3), leaf development (GO:0048366) and (4) leaf morphogenesis (GO:0009965) (Figure 4B). We also identified a few processes that are not overrepresented but include key candidate genes: (5) L-phenylalanine metabolism (GO:0006558), (6) carbohydrate transport (GO: 0034219), (7) lipid transport (GO:0006869), (8) transmembrane transport (GO:0055085), (9) wax biosynthesis (GO:0006633) and (10) fatty acid biosynthesis (GO:0010025) (Figure 4B). Altogether our results suggest that pseudoflower development involves extensive reprogramming of shoot and leaf development, synthesis of volatiles, and changes to the host cell surface. These modifications are consistent with the phenotypes noted in pseudoflowers, notably stem elongation, clustering of morphologically altered leaves that are covered by nectar-like substances and that emit a distinct scent [5].

We propose that the differentially regulated biological processes mentioned above constitute key processes involved in the remarkable developmental changes that take place in *P* *. monoica* -induced pseudoflowers. Our data confirms previous observations that suggest *P* *. monoica* manipulates the host to generate novel pseudofloral structures rather than exploiting existing floral machinery [5]. Among the key biological processes related to host cell development and metabolism that are altered in *P* *. monoica* -induced pseudoflowers (‘Pf’) compared to uninfected stems and leaves (‘SL’), we selected 31 candidate genes (20 up-regulated, and 11 down-regulated) and classified them into nine groups for detailed discussion in the following sections (Table 1).

### De-differentiation of infected mesophyll cells

Our findings indicate that in order to alter leaf development and produce pseudoflowers, *P* *. monoica* appears to induce the de-differentiation of host cells. We observed up-regulation of the *TEOSINTE BRANCHED1, CYCLOIDEA*, and *PCF TRANSCRIPTION FACTORS* (*TCP*) *2* (*TCP2*, At4g18390) and *3* (*TCP3*, At1g53230) genes in pseudoflowers (Table 1 and Figure 4B). These transcription factors are involved in maintaining undifferentiated cells in the shoot apical meristem (SAM) and in coordinating differentiation in leaf organs [17,18]. In addition, we noted the down-regulation of *ALTERED MERISTEM PROGRAMMING1* (*AMP1*, At3g54720) that acts to promote cell differentiation [19,20] (Table 1 and Figure 4B). Mutations in *AMP1* in *A. thaliana* leads to increased leaf initiation, reduced leaf and stem size, and apical dominance [19]. Therefore, up-regulation of *TCPs* and down-regulation of *AMP1*, could promote de-differentiation of infected mesophyll cells. De-regulation of these genes may also prevent branching of leaves growing in the upper part of the stem (cauline leaves) by suppression of lateral shoot development, thereby maintaining apical dominance in stems bearing pseudoflowers (Figure 1B).

### Alteration of the rate of cell proliferation

Pseudoflowers consist of modified leaves differing in size and shape relative to uninfected *B* *. stricta* leaves (Figure 1). Cell proliferation and cell expansion processes of leaf morphogenesis are required to produce the final leaf shape [21], and these processes might be altered by *P* *. monoica* to achieve pseudoflower morphogenesis. In accordance, we found up-regulation of the *ROTUNDIFOLIA-LIKE2* (*RTFL2*, At2g29125) gene in pseudoflowers (Table 1 and Figure 4B). Overexpression of *ROTUNDIFOLIA4* (*ROT4*), a homolog of *RTFL2* decreases cell numbers specifically in the leaf-length direction resulting in shortened and rounded leaves in *A. thaliana* [22,23] suggesting that this gene may regulate cell proliferation during pseudoflowers morphogenesis.

### Alteration of coordinated organ growth and symmetry

Many plants have an outcrossing (allogamy) reproduction strategy and their reproductive success depends on pollinators [24]. The size and architecture of floral organs determines flower shape and attractiveness towards insect visitors [24]. Analogous to allogamous plants, the rust fungus *P* *. monoica* also relies on pollinators [5,8]. We observed symmetrically arranged flower-like leaves at the top of the pseudoflowers (Figure 1B), suggesting that *P* *. monoica* alters the growth of individual leaves to produce the characteristic pseudofloral structures. Consistent with this hypothesis, the cytochrome P450 monooxygenase *KLUH/CYP78A5* (At1g13710) gene was up-regulated in pseudoflowers (Table 1 and Figure 4B). *KLUH/CYP78A5* promotes leaf and floral organ growth [25], particularly by coordinating the growth of individual flower organs, and contributing to uniformity of flower size and symmetry [26]. Our results suggest *CYP78A5* may coordinate the symmetry of pseudofloral leaf clusters (Figure 1B), an important visual cue known to attract pollinators [27,28].

### Alteration of vascular patterning and phyllotaxy

The dramatic morphological alterations in pseudoflowers (Figure 1B) imply the establishment of specific vascular bundle and leaf patterns. Several transcription factors controlling these processes were differentially expressed in pseudoflowers: the *INCURVATA4* (*ICU4*, At1g52150) and *PHAVOLUTA* (*PHV*, At1g30490) genes were down-regulated, whereas the *FILAMENTOUS FLOWER* (*FIL*, At2g45190) and *NGATHA3* (*NGA3*, At1g01030) genes were up-regulated (Table 1 and Figure 4B). *ICU4* encodes ATHB-15, an HD-ZIP III transcriptional factor required for shoot apical meristem patterning and stem vascular differentiation [29]. *A. thaliana icu4* mutants display an abnormal arrangement of leaves due to impaired shoot apical meristem development, producing paired leaves along the stem and axillary shoots [29]. *PHV* encodes another HD-ZIP III transcriptional factor involved in specification of the upper (adaxial) surface of leaves [30]. *FIL* is a member of the YABBY family of transcriptional regulators required for vascular differentiation, specifically in the abaxialization of leaves [31]. Transgenic *A. thaliana* plants expressing *FIL* form filamentous leaves with mainly abaxial looking tissues [31]. Over-expression of *NGA3* in transgenic plants results in apical dominance and alters flower phyllotaxy. It also causes abnormal arrangement of leaves in the stem axis, longer, darker, and narrower rosette leaves, as well as a flattened stem [32]. Overall, the down-regulation of master transcriptional regulators of leaf development, *ICU4, PHV* and up-regulation of *FIL* and *NGA3* could contribute to the altered vascular pattering and morphology of leaves in pseudoflowers [5] (Figure 1B).

### Inhibition of flower differentiation and maturation

The formation of pseudoflowers is likely to involve the inhibition of floral signals and floral organ development in the host. Accordingly, five genes involved in the flowering transition were differentially expressed in pseudoflowers: *FLOWERING LOCUS T* (*FT*, At1g65480), *KNOTTED-LIKE1* (*KNAT1*, At4g08150), *POUND-FOOLISH* (*PNF*, At2g27990) and *SEPATALLA4* (*SEP4*, At2g03710) genes were down-regulated, whereas the *QUARTER2* (*QRT2*, At3g07970) gene was up-regulated (Table 1 and Figure 4B). *FT* produces a mobile floral activator signal protein that moves through the phloem from induced leaves to the shoot apex where it interacts with the FLOWERING LOCUS D (FD) bZIP transcription factor to initiate transcription of floral specification genes [33,34]. Down-regulation of *FT* in pseudoflowers suggests interference with the activation and transmission of floral cues, probably leading to inhibition of floral organ development in infected plants (Figure 1B). Loss of *KNAT1*, a member of the *class1 Knotted1-like homeobox* (*KNOX*) family of transcriptional regulators, results in reduced growth of floral pedicels, internodes and the style during reproductive growth [35,36]. *PNF* and its paralog *PENNYWISE* (*PNY*, At5g02030) encode for BEL1-like homeobox (BLH) proteins that regulate inflorescence internode patterning [37,38] and are also required for floral formation mediated by *FT* [39]. *Arabidopsis* *pny pnf* double mutants initiate compact shoots that fail to respond to flowering signals and subsequently never form flowers [37]. Together with other MADS-box transcription factors, *SEP4* plays a central role in floral meristem and floral organ identity [40]. *A. thaliana sep4* single mutants do not exhibit visible phenotypes, but when all four members of the *SEP* gene family (*sep1 sep2 sep3 sep4*) are mutated, plants show a conversion of floral organs to leaf-like organs [40]. As opposed to these four transcription factors, *QRT2*, which encodes a polygalacturonase involved in cell division, was up-regulated in pseudoflowers (Table 1 and Figure 4B). Plants over-expressing *QRT2* have flowers that do not open, atypical petals, and anthers that fail to dehisce normally [41]. Together these results suggest that the regulation of several genes involved in floral organ differentiation and maturation could potentially act in conjunction to inhibit flower formation in infected plants (Figure 1B). We hypothesize that inhibition of flowering could prolong the lifespan of *P* *. monoica* infected plants, benefiting the parasite at the expense of host plant fitness and reproductive success [5].

### Alteration of auxin homeostasis

Pseudoflowers consist of clusters of elongated stems that bolt from infected rosettes and almost never reach flowering. Changes in the regulation of plant host hormones involved in organogenesis may contribute to the formation of these dense flower-like clusters. Accordingly, we found that genes involved in various mechanisms that control auxin homeostasis were up-regulated in pseudoflowers. Among these are the *TRYPTOPHAN AMINOTRANSFERASE OF ARABIDOPSIS1* (*TAA1*, At1g70560) gene that is essential for Trp-dependent indole-3-acetic acid (IAA) biosynthesis [42] and the *IAA-AMIDO SYNTHASE2* (*GH3.2*, At4g37390) and *4* (*GH3.4*, At1g59500), which are involved in the production of IAA conjugates to regulate the level of active auxin inside the plant [43] (Table 1 and Figure 4B). Also, we identified two genes that were up-regulated as involved in auxin-transport and auxin-mediated organogenesis processes: *P-GLYCOPROTEIN2* (*PGP2*, At4g25960) that encodes a protein with homology to PGP1 known to mediate hypocotyl growth [44,45] and *protein serine/threonine AGC KINASE WAG2* (At3g14370) that positively regulates cotyledon formation [46] (Table 1 and Figure 4B). Up-regulation of genes involved in auxin-mediated organogenesis could contribute to stem elongation and growth of leaves in pseudoflowers [5] (Figure 1B).

### Activation of wax biosynthesis and cutin transport

Plants under water deficit show decreased stem elongation [47] and restricted formation and number of leaves due to increased leaf senescence [48]. To help prevent water loss under stressful conditions some plants secrete and accumulate waxes in the surface of the leaves [49]. We found in pseudoflowers the up-regulation of key genes involved in wax and cutin biosynthesis and transport in *A. thaliana* leaves and stems: *WDS7* (At5g12420), a homolog of the *Wax Ester Synthase/AcylCoA*:*diacylglycerol acyltransferase1* (*WSD1*), 3-*ketoacyl-CoA synthase8* (*KCS8*, At2g15090), and *DEFECTIVE IN CUTICULAR RIDGES/PERMEABLE LEAVES3* (*DCR/PEL3*, At5g23940) (Table 1 and Figure 4B). In *A. thaliana*, *WSD7* homolog, *WSD1*, is a wax synthase required for stem wax ester biosynthesis [50], *KCS8* is a component of the fatty acid elongase complex required for the synthesis of epicuticular waxes in leaves [51], and *DCR* encodes a putative acyltransferase required for the incorporation of the monomer 9(10),16-dihydroxy-hexadecanoic acid into the cutin polymeric structure of young expanding leaves and flowers [52]. In addition, we observed the up-regulation of *ATP-binding-cassette (ABC*)* transporters superfamily G13* (*ABCG13*, At1g51460) (Table 1 and Figure 4B) that mediates secretion and transport of cuticular lipids in flower organ surfaces [53]. Interestingly, *ABCG13* expression and phenotypes have only been detected in true flowers [53], indicating that *P* *. monoica* induces flower-like wax metabolism in stems and leaves.

The up-regulation of wax biosynthesis genes *KCS8, DCR* and *WSD7*, and cutin transport *ABCG13*, points to changes in wax production and cutin allocation in pseudoflowers. Changes in wax composition could positively affect development and longevity of leaves and potentially benefit *P* *. monoica* by maintaining the nutrient supply. Alterations in wax composition could also improve rust spore adhesion during subsequent fertilization given that the cuticular lipid constituents of plant surfaces are known to affect germination and appressorium formation of fungal spores [54].

### Subversion of sugar metabolism

We noted that the expression of several *B* *. stricta* genes involved in sugar metabolism is altered in pseudoflowers. Two *SWEET* genes encoding sugar transporters, *SWEET1* (At1g21460) and *SWEET15* (At5g13170) [55], are up-regulated (Table 1 and Figure 4B), suggesting that sugar transporters might be co-opted during infection by *P* *. monoica* for nutritional gain. *CELL wall invertase1* (*cwINV1*, At3g13790) (Table 1 and Figure 4B), which in *A. thaliana* is induced upon fungal infection [56], is also up-regulated. Cell wall invertases control plant metabolism by hydrolyzing sucrose and providing apoplastic glucose and fructose to the cells [57]. Among the *A. thaliana* cell wall invertase family, *cwINV1* and its paralog *cwINV4*, which are required for sugar accumulation during nectar production [58], are highly expressed in flowers [59]. We hypothesize that up-regulation of *cwINV1* could contribute to the production of the nectar-like substance over the surface of pseudoflowers (Figure 1B). Nectar is the principal floral reward for pollinators [60], and its production in pseudoflowers should benefit the rust pathogen [5]. Unlike the flower-expressed *cwINV4*, *cwINV1* is highly expressed in leaves [59], perhaps facilitating manipulation by the rust fungus.

Conversely, *INDETERMINATE14* (*IDD14*, At1g68130) and *SUCROSE SYNTHASE4* (*SUS4*, At5g20830) are down-regulated in pseudoflowers (Table 1 and Figure 4B). IDD14 shares homology with IDD8, an *A. thaliana* protein with a zing finger ID-domain (IDD) that indirectly promotes flowering via transcriptional activation of *SUS4* [61]. Activation of *SUS4* regulates photoperiodic flowering through the modulation of sugar metabolism and transport [62]. *IDD14* and *SUS4* down-regulation therefore suggesting transcriptional modulation of host sugar metabolism leading to the repression of flowering, that may prolong the infected plant vegetative phase and favour *P* *. monoica* .

### Alteration of volatile organic compounds synthesis

**Figure 5:**
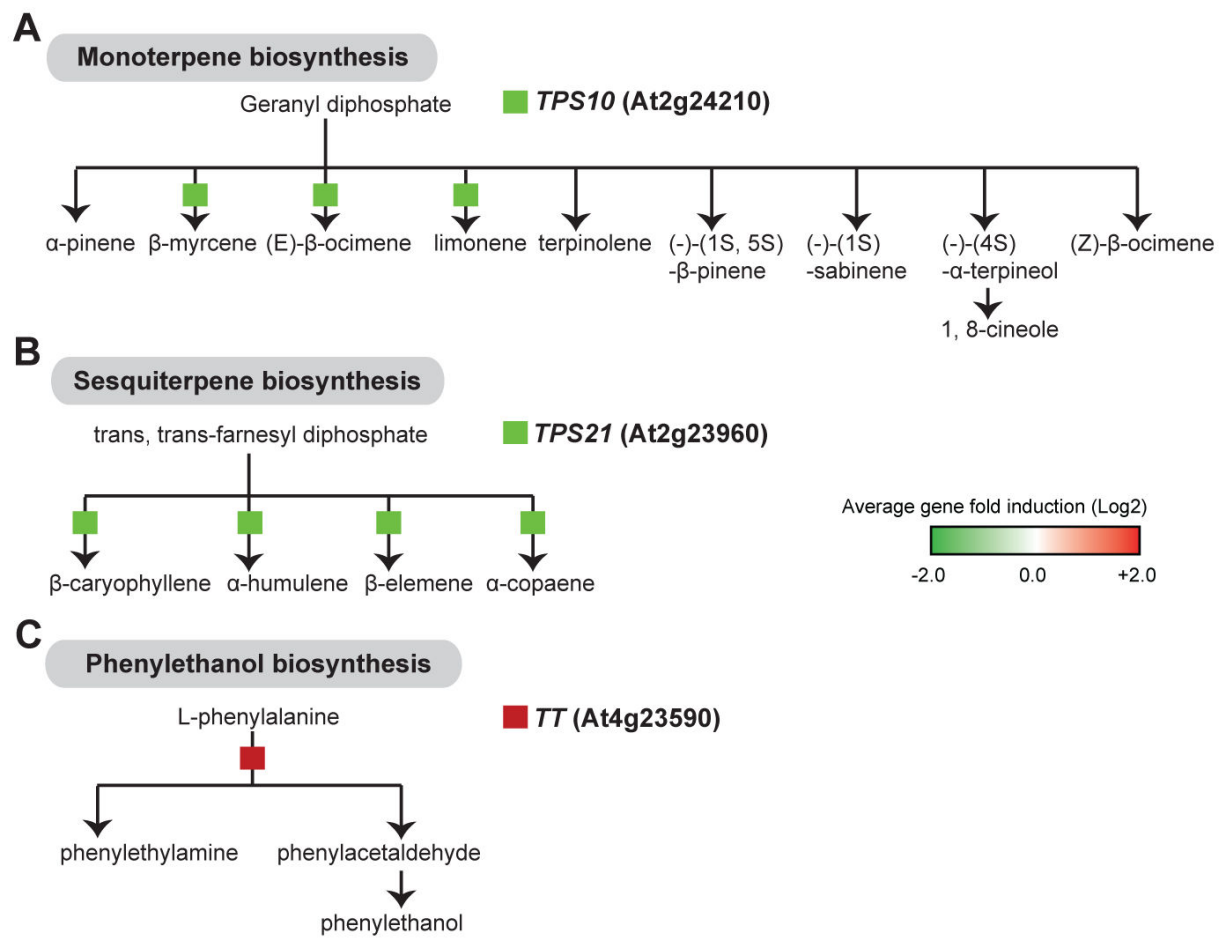
Altered expression in pseudoflowers of genes involved in the biosynthesis of aromatic compounds. (**A**) Down-regulation of genes involved in terpene (monoterpenes and sesquiterpenes) biosynthetic pathway: *TERPENE* *SYNTHASE10* (*TPS10*, At2g24210) and *21* (*TPS21*, At2g23960). (**B**) Up-regulation of genes involved in phenylethanol biosynthetic pathway: *TYROSINE* *TRANSAMINASE* enzyme encoding gene (*TT*, At4g23590). Pathway diagrams were obtained from AraCyc and PlantCyc browsers (http://plantcyc.org/). Blocks represent genes involved in the production of particular compounds within the metabolic pathway. The color of the block indicates relative gene fold induction (from green for average induction folds < 0 that indicates down-regulation to red for average induction folds > 0 that indicates up-regulation) in *Pucciniamonoica* -induced pseudoflowers (‘Pf’) compared to uninfected *Boechera* *stricta* stems and leaves (‘SL’).

The attraction of insect pollinators by pseudoflowers involves volatile compounds that significantly differ from the host flower fragrances [10,11]. The fragrance of *Arabidopsis* spp. flowers is predominantly composed of monoterpenes and sesquiterpenes volatile organic compounds (VOCs) [63]. We identified two genes involved in terperne biosynthesis in *Arabidopsis* , *TERPENE SYNTHASE10* (*TPS10*, At2g24210) and 21 (*TPS21*, At5g23960), that were down-regulated in pseudoflowers (Table 1, Figure 4B and Figure 5A-B). *TPS10* encodes a β-myrcene/(*E*)-β-ocimene synthase normally expressed in flowers and leaves [63,64] and *TPS21* encodes a α-humulene/(-)-(E)-β-caryophyllene synthase expressed almost exclusively in flowers [63,65]. Down-regulation of these two *TPS* genes suggests the absence of terpene blends in pseudoflowers as shown by Raguso and Roy [10].

In contrast one gene involved in phenylalanine degradation was up-regulated: Tyrosine transaminase (TT, At4g23590) (Table 1, Figure 4B and Figure 5C). This pathway ultimately produces the VOCs phenylacetaldehyde and phenylethylethanol. This finding is consistent with a previous study that identified phenylacetaldehyde and phenylethylethanol as the most abundant volatiles in *Puccinia*-induced pseudoflowers [10]. Phenylacetaldehyde is a well-known attractant of foraging insects [66] and has been proposed to attract pollinators in certain *Arabidopsis* ecotypes [67]. Our results are therefore consistent with the hypothesis by Raguso and Roy [10] that pseudoflowers produce fragrances by modifying host-plant metabolites.

## Conclusions

Using whole-genome expression profiling, we identified a large number of plant genes that with altered expression in *P* *. monoica* -induced pseudoflowers (‘Pf’) compared to uninfected *B* *. stricta* stems and leaves (‘SL’). We report nine major host processes related to cell development (de-differentiation of mesophyll cells, rate of cell proliferation, coordinated organ growth and symmetry, of vascular pattering and inhibition of floral organs) and to host metabolism (auxin homeostasis, host cell wax surface compound synthesis, sugar metabolism and volatile organic compounds synthesis) that appear to be re-programmed at the transcriptional level in pseudoflowers. Alterations in the expression of regulators of cell fate and auxin homeostasis genes may account for the distinctive morphology of pseudoflowers, which are essentially modified leaves [5]. Up-regulation of genes controlling stem and leaf development and individual organ symmetry may contribute to the characteristic phenotype in pseudoflowers (infected plants having elongated stems bearing modified short leaves with symmetrically arranged flower-like leaves at the top) (Figure 1B). Down-regulation of genes involved in floral organ development and floral transition may prevent the switch from the vegetative phase to flowering. Genes associated with cuticular wax production that are up-regulated in pseudoflowers could help to protect and maintain leaf longevity in stressful conditions and this could benefit *P* *. monoica* nutrient supply. Up-regulation of genes involved in sugar metabolism and transport could provide carbon sources for *P* *. monoica* , and contribute to the synthesis of nectary substances attractive to pollinators. Finally, down-regulation of terpene VOCs synthesis genes and up-regulation of phenylacetaldehyde synthesis may contribute to the distinct pseudoflower fragrance resulting in odorant cues to attract insects.

Our expression profiling experiments of *P* *. monoica* -infected pseudoflowers has provided a number of new insights into how obligate fungal pathogens manipulate both their plant and insect hosts. Our findings contribute significant insight into the dramatic morphological and physiological changes that occur in *B* *. stricta* plants infected by this rust fungus.

## Materials and Methods

### Plant material and RNA extraction

In this study we collected naturally *Pucciniamonoica* -infected *Boechera* *stricta* plants for gene expression analysis. Artificially infection of *B* *. stricta* plants with *P* *. monoica* in the laboratory is not feasible as its alternate host grass, on which *P* *. monoica* must grow to complete its life cycle, is unknown. We extracted total RNA from *P* *. monoica* -induced pseudoflowers (‘Pf’) (3 samples), uninfected *B* *. stricta* plant stems and leaves (‘SL’) (3 samples), and uninfected *B* *. stricta* flowers (‘F’) (2 samples). Tissue was collected in Gothic (2900 m) (Colorado, USA), and stored in RNA*later*® tissue collection solution (Invitrogen, Cat No. AM7020). Total RNA was extracted using TRIzol® Reagent (Invitrogen, Cat No. 15596-026) according to the manufacturer’s instructions. RNA quality and integrity were assessed prior to cDNA synthesis using the Bioanalyzer (Agilent 2100). NimbleGen microarray services were used for cDNA preparations, chip hybridizations to an *Arabidopsis thaliana* NimbleGen array design 4 x 72K (4-plex format with 72000 probes, two probes per target gene and a total of 30362 target genes, Cat No. A4511001-00-01) and subsequent normalization of the probe sets using Robust Multichip Average (RMA) [68].

### Ethics statement

Samples were collected from Gothic in a location that is about 5 miles away from the Rocky Mountain Biological Laboratory (RMBL) near Gunnison (Colorado, USA). The location used in this study is not private owned or protected and has been previously reported for sampling of *Boechera* *stricta* [69]. *B* *. stricta* is not a protected plant species in Colorado, USA. No specific permissions (for both the location and plant material) were required for the collection of the samples.

### Gene expression analysis

For the analysis of microarray data, we estimated a False Discovery Rate (FDR) for differential gene expression using the Rank Products (RP) program [15]. This program performs permutations with no data distribution assumptions and is recommended for samples obtained outside of controlled laboratory conditions [70]. We applied the RP analysis with 5000 permutations on two comparisons of samples: (i) *Pucciniamonoica* -induced pseudoflowers ‘Pf’ vs. uninfected *Boechera* *stricta* plant stems and leaves ‘SL’ and (ii) uninfected *B* *. stricta* flowers ‘F’ vs. ‘SL’. A threshold of RP FDR value < 0.05 was used to identify differentially regulated genes (up-regulated and down-regulated) in each comparison.

### Accession numbers

The NimbleGen microarray data used in this publication have been deposited on the Gene Expression Omnibus (GEO) (GEO, http://www.ncbi.nlm.nih.gov/geo/) and are accessible through GEO series accession number GSE41165.

### Quantitative real time PCR

Seven candidate *Arabidopsis thaliana* genes that exhibited changes in expression in *Pucciniamonoica* -induced pseudoflowers (‘Pf’) compared to uninfected *Boechera* *stricta* stems and leaves (‘SL’) in the microarray experiment were selected for validation via quantitative real time PCR (qRT-PCR). Primers were designed wherever possible to anneal at 60°C and to either sit on or amplify across an exon boundary to avoid contamination from genomic DNA using amplify v3.1 software (© Bill Engels, University of Wisconsin) (see Table S5). All amplicons were initially confirmed by agarose electrophoresis to determine if the amplicon was the predicted size and a single product. The qRT-PCR analyses were carried out in a CFX96™ real-time PCR system (Bio-Rad) instrument using the SYBR Green JumpStart Taq ready mix (Sigma, Cat No. S4438) on cDNA samples produced with the SuperScript III first-strand synthesis system kit (Invitrogen, Cat No. 18080-051) following the manufacturer’s instructions. The qRT-PCR experiments were performed with cDNAs of three tissues: pseudoflowers (‘Pf’), *B* *. stricta* flowers (‘F’) and *B* *. stricta* stems and leaves (‘SL’). To quantify the relative expression level of a particular gene, three independent qRT-PCR reactions (technical replicates) were performed on biological duplicate samples for each tissue analyzed. All data points were normalized to the internal control gene *ELONGATION FACTOR1 ALPHA* (*EF-1alpha*, At1g18070) and relative to samples collected *B* *. stricta* stems and leaves (‘SL’) using the comparative CT (ΔΔCT) method. ΔCT is defined as the difference in the cycle threshold (CT) between the gene of interest and the *EF-1alpha* control. Relative expression levels were calculated as the log_2_ ratio = log_2_ [(ΔCT of ‘Pf’ at gene X)/(ΔCT of ‘SL’ at gene X)], with ‘X’ corresponding to the gene of interest. The statistical T-test was performed using R software to determine differences between ‘Pf’ vs. ‘SL’ and ‘F’ vs. ‘SL’ group. A *P*-value < 0.05 was defined as statistically significant. To indicate the mode of regulation we used two symbols: ‘*’ for significant up-regulation and ‘^#^’ for significant down-regulation. The number of symbols indicates level of significance: one for *P* < 0.05, two for *P* < 0.01 and three for *P* < 0.001. Data is presented as average ± SEM.

### Gene Ontology (GO) enrichment and pathway analysis

A list of GO annotations for *Arabidopsis thaliana* was extracted from The *Arabidopsis* Information Resource (TAIR) database [71]. Using the BiNGO plug-in available for Cytoscape [16], over-represented groups of GO terms and functional domains were identified using a hypergeometric test with Benjamin & Hochberg False Discovery Rate (FDR) correction and a *P*-value threshold of 0.05. This test identified significantly enriched GO categories by comparing *Arabidopsis thaliana* 27822 GO annotated genes with the 1036 and 910 genes that showed significant changes in gene expression in (i) *Pucciniamonoica* -induced pseudoflowers ‘Pf’ vs. uninfected *Boechera* *stricta* stems and leaves ‘SL’ and (ii) uninfected *B* *. stricta* flowers ‘F’ vs. ‘SL’ comparisons, respectively. Using Cytoscape visualization tools we constructed a network map to illustrate significantly enriched Gene Ontology terms describing Biological Processes (GOBP) in (i) ‘Pf’ vs. ‘SL’ and (ii) ‘F’ vs. ‘SL’ comparisons, respectively. The size of the node in the network map corresponds to -log_10_ of the corrected *P*-value of enrichment within a GOBP term. In addition, snapshots of *A. thaliana* AraCyc and PlantCyc browsers (http://plantcyc.org/) were used to visualize specific metabolic pathways that were significantly regulated in ‘Pf’ vs. ‘SL’.

## Supporting Information

Table S1 **List of 1036 significantly differentially expressed genes in pseudoflowers.** 'Pf': *Pucciniamonoica* -induced pseudoflowers. 'SL': *Boechera* *stricta* uninfected stems and leaves. *False Discovery Rate (FDR) estimated using Rank Products (RP) to detect genes that are differentially expressed. Genes with RP FDR value < 0.05 are considered significant.
(XLSX)

Table S2 **List of 910 significantly differentially expressed genes in uninfected *Boechera* *stricta* flowers.** 'F': *Boechera* *stricta* uninfected flowers. 'SL': *Boechera* *stricta* uninfected stems and leaves. *False Discovery Rate (FDR) estimated using Rank Products (RP) to detect genes that are differentially expressed. Genes with RP FDR value < 0.05 are considered significant.
(XLSX)

Table S3 **List of 256 gene ontology biological processes (GOBP) enriched in pseudoflowers.** 'Pf': *Pucciniamonoica* -induced pseudoflowers. 'SL': *Boechera* *stricta* uninfected stems and leaves. ^a^Number of genes within found within the biological process. ^b^P-value showing the significance for enrichment of genes within the biological process.
(XLSX)

Table S4 **List of 199 gene ontology biological processes (GOBP) enriched in uninfected *Boechera* *stricta* flowers.** 'F': *Boechera* *stricta* uninfected flowers. 'SL': *Boechera* *stricta* uninfected stems and leaves. ^a^Number of genes within found within the biological process. ^b^P-value showing the significance for enrichment of genes within the biological process.
(XLSX)

Table S5 **Primer sequences used for qRT-PCR assay.**
(XLSX)
